# Myocardial Chemokine Expression and Intensity of Myocarditis in Chagas Cardiomyopathy Are Controlled by Polymorphisms in *CXCL9* and *CXCL10*

**DOI:** 10.1371/journal.pntd.0001867

**Published:** 2012-10-25

**Authors:** Luciana Gabriel Nogueira, Ronaldo Honorato Barros Santos, Barbara Maria Ianni, Alfredo Inácio Fiorelli, Eliane Conti Mairena, Luiz Alberto Benvenuti, Amanda Frade, Eduardo Donadi, Fabrício Dias, Bruno Saba, Hui-Tzu Lin Wang, Abilio Fragata, Marcelo Sampaio, Mario Hiroyuki Hirata, Paula Buck, Charles Mady, Edimar Alcides Bocchi, Noedir Antonio Stolf, Jorge Kalil, Edecio Cunha-Neto

**Affiliations:** 1 Laboratory of Immunology, Heart Institute (InCor), School of Medicine, University of São Paulo, São Paulo, Brazil; 2 Division of Clinical Immunology and Allergy, School of Medicine, University of São Paulo, São Paulo, Brazil; 3 Institute for Investigation in Immunology (iii), INCT, São Paulo, Brazil; 4 Divison of Surgery, Heart Institute (InCor), School of Medicine, University of São Paulo, São Paulo, Brazil; 5 Myocardiopathies Unit, Heart Institute (InCor), School of Medicine, University of São Paulo, São Paulo, Brazil; 6 Divison of Pathology, Heart Institute (InCor), School of Medicine, University of São Paulo, São Paulo, Brazil; 7 School of Medicine of Ribeirão Preto (FMRP), University of São Paulo, São Paulo, Brazil; 8 Dante Pazzanese Institute of Cardiology and Heart Failure Unit, São Paulo, Brazil; 9 Transplantation and Heart Failure Unit, Heart Institute (InCor), School of Medicine, University of São Paulo, São Paulo, Brazil; Federal University of São Paulo, Brazil

## Abstract

**Background:**

Chronic Chagas cardiomyopathy (CCC), a life-threatening inflammatory dilated cardiomyopathy, affects 30% of the approximately 8 million patients infected by *Trypanosoma cruzi*. Even though the Th1 T cell-rich myocarditis plays a pivotal role in CCC pathogenesis, little is known about the factors controlling inflammatory cell migration to CCC myocardium.

**Methods and Results:**

Using confocal immunofluorescence and quantitative PCR, we studied cell surface staining and gene expression of the CXCR3, CCR4, CCR5, CCR7, CCR8 receptors and their chemokine ligands in myocardial samples from end-stage CCC patients. CCR5+, CXCR3+, CCR4+, CCL5+ and CXCL9+ mononuclear cells were observed in CCC myocardium. mRNA expression of the chemokines CCL5, CXCL9, CXCL10, CCL17, CCL19 and their receptors was upregulated in CCC myocardium. CXCL9 mRNA expression directly correlated with the intensity of myocarditis, as well as with mRNA expression of CXCR3, CCR4, CCR5, CCR7, CCR8 and their ligands. We also analyzed single-nucleotide polymorphisms for genes encoding the most highly expressed chemokines and receptors in a cohort of Chagas disease patients. CCC patients with ventricular dysfunction displayed reduced genotypic frequencies of *CXCL9* rs10336 *CC*, *CXCL10* rs3921 *GG*, and increased *CCR5* rs1799988*CC* as compared to those without dysfunction. Significantly, myocardial samples from CCC patients carrying the *CXCL9/CXCL10* genotypes associated to a lower risk displayed a 2–6 fold reduction in mRNA expression of CXCL9, CXCL10, and other chemokines and receptors, along with reduced intensity of myocarditis, as compared to those with other *CXCL9/CXCL10* genotypes.

**Conclusions:**

Results may indicate that genotypes associated to reduced risk in closely linked *CXCL9* and *CXCL10* genes may modulate local expression of the chemokines themselves, and simultaneously affect myocardial expression of other key chemokines as well as intensity of myocarditis. Taken together our results may suggest that CXCL9 and CXCL10 are master regulators of myocardial inflammatory cell migration, perhaps affecting clinical progression to the life-threatening form of CCC.

## Author Summary

Chronic Chagas cardiomyopathy (CCC) is an inflammatory heart disease that affects millions in Latin America, and in growing numbers in USA and Europe. Survival among CCC patients is shorter than among patients with cardiomyopathy of non-inflammatory etiology. This suggests that the inflammatory cell influx plays an important pathogenic role in CCC. However, little is known about the factors that maintain this myocardial inflammation. We hypothesized that Th1 T cell-attracting chemokines, involved in driving leukocyte migration, could play a role in myocardial inflammation. Herein, we have analyzed expression of several chemokines and receptors in heart tissue from patients with CCC and controls. We found inflammatory cells expressing chemokines and receptors consistent with Th1 T cell influx into CCC myocardium. mRNA expression levels of the chemokine CXCL9 correlated with inflammation. We also studied whether genetic variations in these genes could be associated to CCC development. Polymorphisms in *CXCL9*, *CXCL10* and *CCR5* were associated to differential risk of progression to the more severe form of CCC. Polymorphisms of *CXCL9* and *CXCL10* were also associated to the intensity of myocardial inflammation and chemokine expression. These results suggest that such chemokines may be master regulators of myocardial inflammatory cell migration, perhaps affecting clinical progression to severe CCC.

## Introduction

Approximately 8 million people are infected with the protozoan parasite *Trypanosoma cruzi* [1] in Central and South America, with an estimated 300,000 cases in the USA alone. *T. cruzi* is a major cause of heart disease and cardiovascular-related deaths in endemic areas located in Latin America, with approximately 50,000 fatalities per year due to Chronic Chagas cardiomyopathy (CCC) [2]. The high parasite load typical of the acute infection results in a strong innate and adaptive immune response against *T. cruzi*, leading to the control - but not the complete elimination - of tissue and blood parasitism, establishing a low-grade chronic persistent infection [3]. CCC is an inflammatory cardiomyopathy that affects approximately 30% of infected individuals and occurs 5–30 years after acute infection, while the remaining patients develop digestive disorders (5–10%) or remain asymptomatic (ASY, 60–70%) [4]. It has been observed that the occurrence of myocarditis is correlated with clinical severity, ASY patients having minimal inflammation, while patients with advanced CCC display frequent and intense myocarditis [5]. Approximately 1/3 of patients developing CCC present a particularly lethal form of dilated cardiomyopathy, with shorter survival than idiopathic dilated cardiomyopathy [6]. In addition, CCC is associated with a worse prognosis and survival than other cardiomyopathies of non-inflammatory etiology (NIC) [7]. Although currently used trypanocidal drugs are effective in the treatment of acute or recent infection in children their efficacy in halting the progression of cardiac lesions has not been established yet [8]. Data suggest that the mononuclear inflammatory infiltrate, associated with cardiomyocyte destruction and fibrosis observed only in CCC myocardium, plays a major role in the development and progression of the disease [5], [9].

Histologically, CCC myocardium displays a diffuse myocarditis with focal aspects; a mononuclear infiltrate, intense heart fiber damage, prominent fibrosis and scarcity of *T. cruzi* parasites (reviewed in [10]). The inflammatory infiltrate of CCC heart lesions is composed mainly by T cells and macrophages [9], [11]. Heart-infiltrating T cells and other mononuclear cells predominantly produce TNF-α and IFN-γ [12], [13], [14], and a similar increase in IFN-γ and TNF-α is seen in cardiac tissue from animals infected with *T. cruzi* [15], [16]. An increased number of IFN-γ-producing cells is found in peripheral blood mononuclear cells (PBMC) of CCC patients [14], [17]. Together, data suggest a predominance of Th1-type T cells in CCC myocardium and peripheral blood [18]. However, the factors that determine the migration and accumulation of the Th1-type T cell in CCC heart tissue are still obscure. The possibility that chronic myocardial inflammation and tissue damage in CCC are a consequence of recognition of *T. cruzi* antigen on heart tissue must be entertained. However, *T. cruzi* DNA, a surrogate marker of the presence of living parasites, has been equally detected in hearts of CCC and ASY patients [19], [20], indicating that mere parasite presence in the heart is not sufficient for inducing inflammatory tissue damage. Both *T. cruzi*-specific [21] or heart antigen (cardiac-myosin) - specific T cells - crossreactive with *T. cruzi* antigen [22] - have been identified in the myocardial inflammatory infiltrate. Since both cardiac proteins and *T. cruzi* antigen are present in hearts of both CCC and ASY patients, some other factor distinct from antigen availability must control the difference in inflammation and disease progression between Chagas disease patients.

Given the important role of chemokines such as CCL3, CCL4, CCL5 and CXCL9 and CXCL10 in tissue accumulation of CCR5^+^ and/or CXCR3^+^ Th1-type T cells [23], it is possible that the development and maintenance of the tissue-damaging Th1-rich mononuclear infiltrate in CCC myocardium could be a consequence of the *in situ* expression of chemokine ligands to those receptors. Indeed, increased mRNA levels of CC and CXC chemokines have been detected in the heart tissue of *T. cruzi*-infected mice (reviewed in [24], [25]). Additionally, increased numbers of CCR5^+^ CXCR3^+^ CD4^+^ and CD8^+^ T cells producing IFN-γ and/or TNF-α were found in PBMC from CCC patients, as compared with ASY patients [26]. Previous studies from our group showed that gene expression levels of IFN-γ-inducible chemokines CXCL9 and CXCL10 were significantly up-regulated in CCC myocardium [27]. Local production of CXCL9 and CXCL10 by mononuclear infiltrating and stromal cells leads to the recruitment of effector Th1 lymphocytes into inflamed tissues in delayed hypersensitivity reactions (reviewed in [28]). Conversely, functional polymorphisms controlling expression, and loss-of-function deletions in protein-coding regions of genes encoding chemokines and their receptors have been associated with development of several inflammatory and autoimmune diseases [29], [30], [31]. This indicates that chemokine polymorphisms can control organ-specific inflammatory damage -even in the presence of similar amounts of antigen- by means of regulating inflammatory cell influx. We therefore hypothesized that an imbalance at the Th1-associated chemokine-chemokine receptor axis - perhaps of genetic origin - could play a role in the maintenance of the inflammatory infiltrate in CCC patients.

Here, we analyzed cell surface staining and gene expression of the chemokine receptors CXCR3 and CCR5, associated to the Th1 phenotype, and their ligands. Conversely, we also studied the CCR4 and CCR8 receptors, associated to the Th2 phenotype, and their chemokine ligands, as well as the chemokine receptor CCR7, associated to memory phenotypes, and its ligands in myocardial samples form end-stage CCC and non-inflammatory cardiomyopathy patients, as well as control subjects. In addition, we analyzed single-nucleotide polymorphisms (SNPs) for genes encoding 8 such chemokines and receptors in a cohort of Chagas disease patients stratified according to clinical form (ASY, CCC) and presence of ventricular dysfunction among CCC patients.

## Methods

### Ethics Statement

The protocol was approved by the Institutional Review Board of the University of São Paulo School of Medicine (Protocol number 739/2005 and 0324/2009) and written informed consent was obtained from the patients. In the case of samples from heart donors, written informed consent was obtained from their families.

### Patients and sample collection

All Chagas disease patients were considered serologically positive for antibodies against *T. cruzi* on the basis of results of at least 2 of 3 independent tests (EIA [Hemobio Chagas; Embrabio São Paulo], indirect immunofluorescence assay [IFA-immunocruzi; Biolab Merieux], and indirect hemagglutination test [Biolab Merieux]). All Chagas disease patients underwent standard electrocardiography and echocardiography. Echocardiography was performed in the hospital setting using an Acuson Sequoia model 512 echocardiographer with a broad-band transducer. The left ventricular dimensions and regional and global function evaluations were performed using a 2-dimension and M-mode approach, in accordance with the recommendations of the American Society of Echocardiography. Patients with CCC presented with abnormal electrocardiography findings that ranged from typical conduction abnormalities (right bundle branch block and/or left anterior division hemiblock) to severe arrhythmia [32]. A group of patients also presented varying degrees of ventricular dysfunction classified on the basis of left ventricular ejection fraction, with all other causes of ventricular dysfunction/heart failure excluded. All asymptomatic (ASY) subjects had normal echocardiograph and echocardiogram findings, chest radiographs with no evidence of cardiac enlargement, and normal findings of radiographs of the esophagus and colon.

Myocardial left ventricular free wall heart samples were obtained from end-stage heart failure CCC patients (n = 14, positive for antibodies against *T. cruzi* and born in endemic areas for Chagas disease, five males and nine females, mean age 47.2±14.6 years, Table 1) and end-stage heart failure patients with non-inflammatory cardiomyopathies (NIC, n = 8, five patients with idiopathic dilated cardiomyopathy and three patients with Ischemic cardiomyopathy, all seronegative for *T. cruzi*, eight males, mean age 53.3±7.5 years, Table 1). Control adult heart tissue from the left ventricular-free wall was obtained from nonfailing donor hearts (N, n = 6, males, mean age 32.2±12.8 years, Table 1) not used for cardiac transplantation due to size mismatch with available recipients. Hearts were explanted at the time of heart transplantation at the Heart Institute - InCor, University of São Paulo School of Medicine, São Paulo, SP, Brazil. For histological studies, fresh tissues were fixed in buffered formalin solution and paraffin-embedded; for immunofluorescence, fresh samples were maintained in a 30% sucrose solution for approximately 30 min at 4°C; then they were transferred to OCT Tissue Tek freezing medium and immediately frozen in isopentane and stored at −80°C. For mRNA extraction, samples were quickly dissected, and myocardial tissue was frozen in liquid nitrogen and stored at −80°C. For SNP genotyping, genomic DNA was obtained from myocardial left ventricular free wall heart samples using the QIAamp DNA Blood Max Kit (Qiagen, Hilden, Germany) and stored at −20°C.

**Table 1:** Characteristics of patients and control donors whose myocardial samples were used in gene expression studies.

Internal n°	Age	Sex	Disease group	EF	Fibrosis	LVDD	Hypertrophy	Myocarditis	*ANPRelative*	*BNP expression*
CCC patients										
CCC1	15	M	CCC	17	1+	62	Y	1+	5.0	221.5
CCC2	44	F	CCC	21	1+	70	Y	1+	223.6	780.6
CCC3	61	F	CCC	15	1+	76	Y	1+	2.2	51.2
CCC4	23	F	CCC	39	2+	78	Y	2+	55.8	402.6
CCC5	50	M	CCC	11	2+	82	Y	2+	61.0	468.8
CCC6	49	F	CCC	37	3+	77	Y	3+	57.1	268.6
CCC7	49	F	CCC	15	2+	83	Y	3+	15.0	90.8
CCC8	28	M	CCC	21	2+	68	Y	2+	2.6	8.1
CCC9	54	F	CCC	36	2+	62	Y	2+	155.3	828.2
CCC10	58	M	CCC	29	2+	64	Y	2+	88.2	832.6
CCC11	57	M	CCC	29	1+	71	Y	2+	11.8	65.0
CCC12	60	F	CCC	20	2+	72	Y	3+	7.4	264.6
CCC13	61	F	CCC	27	1+	77	Y	0	157.1	1729.1
CCC14	50	F	CCC	23	2+	61	Y	3+	198.5	1844.1
NIC patients										
NIC1	53	M	IDC	19	1+	77	Y	0	53.0	160.1
NIC2	38	M	IDC	16	1+	88	Y	0	510.0	425.2
NIC3	63	M	IDC	38	1+	ND	Y	0	1.2	13.6
NIC4	55	M	IDC	25	3+	51	Y	0	4.4	29.8
NIC5	58	M	IDC	16	2+	99	Y	0	38.3	179.7
NIC6	62	M	IC	37	2+	75	Y	0	4.0	28.1
NIC7	61	M	IC	33	3+	79	Y	0	0.1	0.2
NIC8	52	M	IC	20	3+	62	Y	0	8.8	546.2
Control individuals										
N1	46	M	Normal	ND	0	ND	N	0	-	-
N2	40	M	Normal	ND	0	ND	N	0	-	-
N3	22	M	Normal	ND	0	ND	N	0	-	-
N4	20	M	Normal	ND	0	ND	N	0	-	-
N5	45	M	Normal	ND	0	ND	N	0	-	-
N6	20	M	Normal	ND	0	ND	N	0	-	-

Age (years). M (male). F (female). CCC (Chronic Chagasic Cardiomyopathy). NIC (Non-inflammatory Cardiomyopathy). IDC (Idipathic Dilated Cardiomyopathy). IC (Ischemic Cardiomyopathy). Normal heart donors were subject to ventilator and vasoactive drugs, and had been under life support for an average of 48 hours. Characterization of the samples as myocarditis, fibrosis and hypertrophy; reference values for the presence of myocarditis and fibrosis: 0: absent; 1+: slight; 2+: moderate; 3+: intense; hypertrophy: Y: yes N: no. EF (Ejection Fraction) was always less than 40%. LVDD (left ventricle diastolic diameter) normal reference value: diameter 39–55 mm. Values of mRNA expression (RT-qPCR) of the atrial natriuretic peptide (ANP) and the brain natriuretic peptide (BNP). -, Control samples were used as calibrators in the RT-qPCR reaction and have no relative expression value using the 2^−ΔΔCt^ calculation method as described in Methods.

Blood samples were collected from the patients who were categorized as being ASY (n = 151, 72 males, mean age 50.8±3.6 years, and 79 females, mean age 54.1±3.5 years) or as having CCC (n = 174, 83 males, mean age 50.8±0.5 years, and 91 females, mean age 48.0±6.6 years) on the basis of clinical, radiological, electrocardiographic (ECG), echocardiographic criteria and all of patients were serologically positive for antibodies against *T. cruzi* and born in endemic areas for Chagas disease. All of ASY patients had normal ECG findings and a normal left ventricular ejection fraction (LVEF) at the time of echocardiography, as well as normal findings for chest, esophagus and colon radiography. The CCC patients presented with typical ECG findings (right bundle branch block and/or left anterior division hemiblock and were classified as having moderate (LVEF>40%, n = 79) or severe (LVEF≤40%), n = 95) CCC. Genomic DNA was extracted by the dodecyltrimethyl ammonium bromide/hexadecyltrimethylammonium bromide method and stored at −20°C.

### Histological and Immunofluorescence staining/Confocal analysis

Individual 5-µm sections of paraffin-embedded tissue or cryosections of frozen myocardial fragments were applied to microscope slides. Slides were subjected either to hematoxylin-eosin or immunofluorescence staining. Standard hematoxylin-eosin staining was performed for evaluation of the intensity and location of the inflammatory infiltrate. Slides were evaluated and scored for the intensity of myocarditis, fibrosis and hypertrophy. Slides for immunofluorescence were washed in phosphate-buffered saline (PBS) and blocked with PBS-2% bovine serum albumin. Slides were incubated overnight at 4°C with the primary monoclonal antibodies anti-CD3, anti-CD4, anti-CD8, anti-CCR5, anti-CXCR3, anti-CCR4, anti-CCL5 or anti-CXCL9 (R&D Systems, Minneapolis, MN, USA); after washing, slides were incubated with the secondary antibody, anti-mouse IgG conjugated to AlexaFluor 633 (Invitrogen, Carlsbad, CA, EUA) and 4′,6′-diamidino-2-phenylindole (DAPI, Sigma-Aldrich, Steinhein, Germany) for nuclear staining. Slides were mounted using antifade mounting medium (Hydromount, National Diagnostics, Atlanta, GA, USA). Fluorescent images were acquired using UV/Laser excitation on an LSM/Meta 510 Zeiss microscope, with an oil immersion objetive (63×, 1.25 numerical aperture). For each section, the area with most uniform infiltrate was selected for analysis. Within this inflammatory area, minimums of five fields were acquired. Image analysis and processing were performed using LSM Image Examiner software (Carl Zeiss, Standort Göttingen, Germany). Omission of primary antibody and replacement with non-immune mouse IgG was used to confirm the lack of nonspecific staining.

### RNA isolation, reverse transcription and quantitative real-time polymerase chain reaction (real-time qPCR)

Total RNA was extracted from 5×5×5 mm myocardial samples using the Trizol® method (Life Technologies Inc., Grand Island, NY). The RNA was quantified using NanoDrop Spectrophotometry (Thermo Scientific), and treated with Rnase-free DNase I (USB, Ohio, USA). cDNA was obtained from 5 µg total RNA using Super-script II™ Reverse Transcriptase (Invitrogen, Carlsbad, CA, USA). We designed forward and reverse primers for real-time qPCR assays using the Primer Express software (Applied Biosystems, Foster City, CA, USA; Table S1). Real-time qPCR reactions were carried out in an ABI Prism 7500 Sequence Detection system (Applied Biosystems) using the SYBR Green PCR Master Mix (Applied Biosystems), as described [33]. For all genes, we constructed standard curves and determined the slope to calculate the PCR efficiency. All the samples were tested in triplicate with the GAPDH, previously shown to display little variance among human myocardial tissue samples [27], as the reference gene for normalization of data, and relative expression of each mRNA was calculated with the 2^−ΔΔCt^ method [34], using expression in six normal donor hearts as calibrator.

### SNP genotyping

We searched for polymorphisms in our target genes preferentially in putatively regulatory regions such as 5′ and 3′untranslated regions (UTR) or intronic regions, and which had previously been studied in disease association studies. This was the case of *CXCL9* rs10336, *CXCL10* rs3921, *CCL5* rs2107538 and *CCR5* rs1799988 [35], [36], [37]. In the cases of target genes where no previous study was found - *CCL*4, *CCL17*, *CCL1*9 and *CXCR3* - the selected SNP's (*CCL*4 rs1719153, *CCL17* rs223827, *CCL1*9 rs3136658 and *CXCR3* rs2280964) were located in putatively regulatory sites, with minimum allele frequencies above 0.2 in the CEU (Caucasian) and Yoruba (YRI) populations at the HapMap database (site http://hapmap.ncbi.nlm.nih.gov) (Table S2). Genotyping was performed using the designed assay of the TaqMan allelic discrimination technique (ABI 7500, Applied Biosystem, Foster City, USA) and evaluated according to the manufacturer's instructions. The following SNPs were tested: SNP *CCL4* rs1719153 (part n°. C_12120537_10), *CCL5* rs2107538 (part n°. C__15874407_10), *CCR5* rs1799988 (part n°. C__11988170_10), *CXCL9* rs10336 (part n°. C_486222_10), *CXCL10* rs3921 (part n°. C_497062_10), *CXCR3* rs2280964 (part n°. C_15874773_10), *CCL17* rs223827 (part n°. C_2845028_10) and *CCL19* rs3136658 (part n°. C__25981926_10) *CCL4*, *CXCL9* and *CXCL10* are mapped to the 3′ untranslated region (UTR); CCL5 and CCR5 are mapped to the 5′ UTR; *CCL17*, *CCL19* and *CXCR3* are mapped to intronic regions.

### Statistical analysis

Values of the relative expression of each mRNA in the CCC and NIC groups were compared with the Mann-Whitney U test. Correlation analysis was performed by Spearman's nonparametric correlation test with SPSS version 14.0 software (SPSS, Chicago, III). Associations between patient groups and genotypes or a specific allele were analyzed by the *x^2^* statistical test, along with the relevant odds ratio (OR) and 95% confidence interval (CI). Fisher's exact test was used when at least one value in the contingency table was <5. We considered differences as significant when the p value was less than 0.05 and the CI did not cross 1. Hardy-Weinberg equilibrium (HWE) was determined by comparing the observed number of different genotypes with the expected number under the HWE for the estimated allele frequency.

## Results

### Patient and sample features

**Figure 1:**
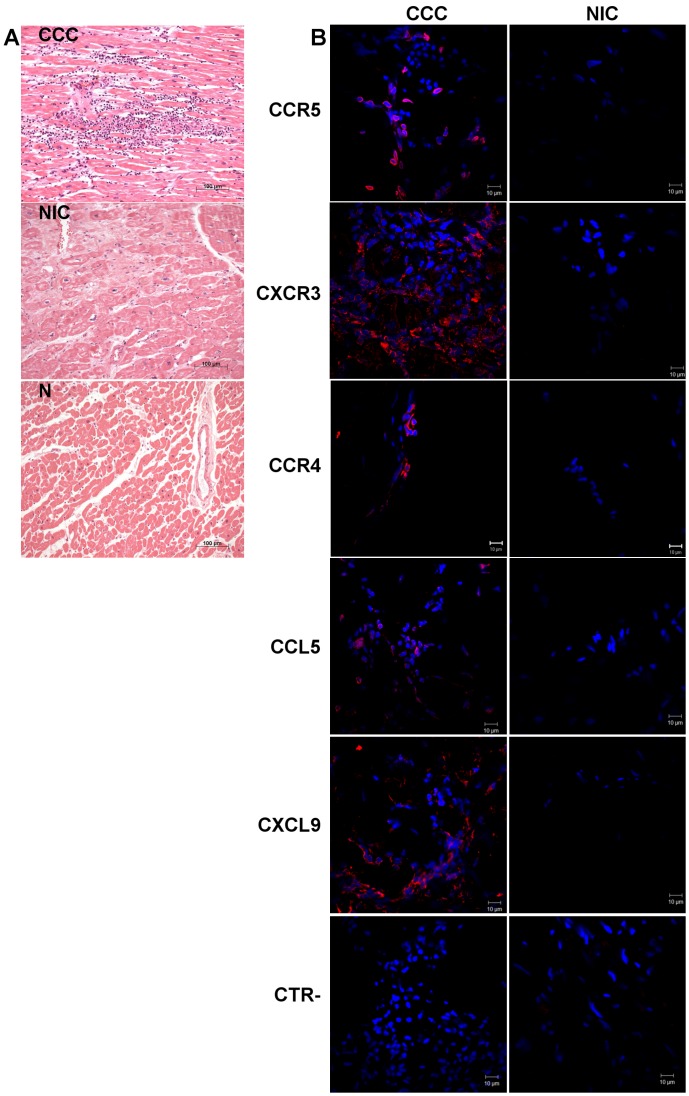
Histopathological analysis and localization of CCR5^+^, CXCR3^+^, CCR4^+^, CCL5^+^ and CXCL9^+^ cells in heart tissue. Severe myocarditis with intense mononuclear inflammatory infiltrate in the heart tissues of CCC patient, and absence of myocarditis in the heart tissues of NIC patients and individuals without cardiomyopathies (N). In (A), imagens representative of heart fragments from CCC, NIC and N individuals. Haematoxylin-eosin stain. Representative images of immunofluorescence confocal microscopy from heart tissue sections from CCC and NIC (B). Sections were stained with primary antibodies against CCR5, CXCR3, CCR4, CCL5 and CXCL9 stained with AF633-conjugated anti-mouse IgG (red) and counterstained with DAPI (blue), as described in Methods. CTR- IgG isotype controls.

While myocardial sections from both CCC and NIC groups displayed cardiomyocyte hypertrophy and fibrosis upon histopathological analysis, lymphocytic myocarditis was only observed among samples from CCC patients (Figure 1A, Table 1). Although all CCC patients presented clinically similar end-stage heart disease, variable degrees of lymphocytic myocarditis were observed. No significant differences were found in age, ejection fraction (EF) or left ventricular diastolic diameter (LVDD) between the two groups, and mRNA expression of the natriuretic peptides ANP and BNP was substantially upregulated in comparison to control myocardium samples (Table 1), indicative of activation of the embryonic/hypertrophic gene expression pattern consistent with advanced heart failure.

### Heart-infiltrating mononuclear express multiple chemokines and chemokine receptors

Using immunofuorescence confocal microscopy, we assessed the surface phenotype of mononuclear cells in the myocardial inflammatory infiltrate of CCC samples (Figure 1B and Figure S1). CD3^+^, CD4^+^ and CD8^+^ T cells were abundant in CCC myocardium, very scarce in NIC samples and essentially absent in control samples (N), in line with histopathological data (Figure S1). We observed mononuclear cells staining for CXCR3 and CCR5-typical of Th1 T cells - as well as CCR4 – more commonly described as a marker of Th2 T cells in CCC myocardium (Figure 1B). CXCR3 staining was also observed in non-mononuclear, stromal cells in CCC heart tissue (Figure 1B, Figure S1). In addition, we observed in situ expression of the chemokines CCL5 and CXCL9, ligands of the CCR5 and CXCR3 receptors, in mononuclear cells infiltrating the myocardium of CCC patients (Figure 1B). We could also observe CCL5 staining in endothelial cells lining what seems to be a blood vessel in CCC myocardium (Figure 1B, Figure S1). Specificity of the tested antibody staining is shown at Figure 1B.

### Multiple T cell chemoattractant chemokines and their receptors are expressed in heart tissue from CCC patients

**Figure 2:**
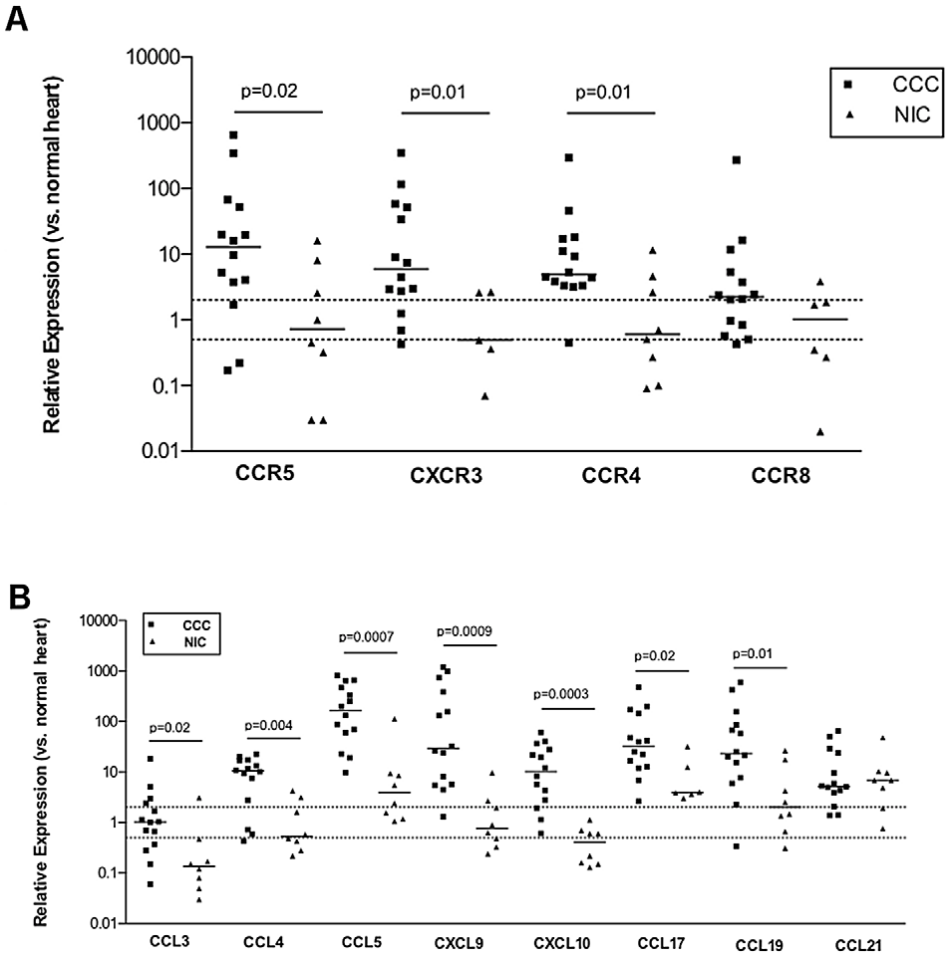
Expression of chemokines and chemokine receptors in myocardium. Expression of chemokine receptors (A) in CCC and NIC myocardium. Expression of chemokines (B) in CCC and NIC myocardium. Real-time qPCR analysis of mRNA levels expression in CCC and NIC myocardium. After normalization to GAPDH mRNA, the relative expression was plotted in comparison to N group and data were calculated with the 2^−ΔΔCt^ method, as described in Methods. The horizontal bar stands for the median. Dotted lines indicate two-fold increase or decrease expression as compared with the control group.

We assessed mRNA expression of CCR5 and CXCR3 chemokine receptors associated with inflammatory, Th1-type T cells, as well as the chemokine receptors CCR4 and CCR8, which are associated to a Th2 phenotype, and CCR7, a chemokine receptor associated to memory phenotypes, in myocardium tissue from CCC, NIC and control samples using real-time qPCR. Figure 2A shows that mRNA expression levels of CCR5, CXCR3 and CCR4 were significantly higher in CCC myocardial tissue that of NIC patients and normal donors, which corroborated with the immunofluorescence detection data (Figure 1B). The expression of CCR8 mRNA did not differ significantly between the CCC and NIC groups (Figure 2A). The expression of CCL1 (CCR8 ligand) was undetectable in CCC, NIC and control samples.

The expression of mRNA encoding CCR5 ligands (CCL3, CCL4 and CCL5), CXCR3 ligands (CXCL9 and CXCL10), the CCR4 ligand CCL17 and the CCR7 ligand CCL19 was significantly higher in CCC than in NIC myocardium (Figure 2B). Of note, median CCL5 expression in CCC myocardium was over 100-fold higher than normal controls. The expression of CXCL9, CXCL10, CCL17 and CCL19 in CCC samples was between 10 and 100-fold higher than normal control samples. Of interest, mRNA levels of CCL21, another CCR7 ligand, were 5-fold higher in samples from both CCC and NIC heart tissue than in samples from control individuals (Figure 2B). As the expression of CCR7 was undetectable in myocardium samples from control individuals, the relative expression of this receptor could not be subjected to relative quantification using the ΔΔCt method. We found that the average ΔCt value of CCR7 in heart tissue from the CCC patients was significantly lower than in NIC myocardium (14.52±2.9 versus 18.34±2.5; p = 0.01, data not shown), indicating that this gene also displayed increased expression in CCC as compared to NIC myocardium.

**Figure 3:**
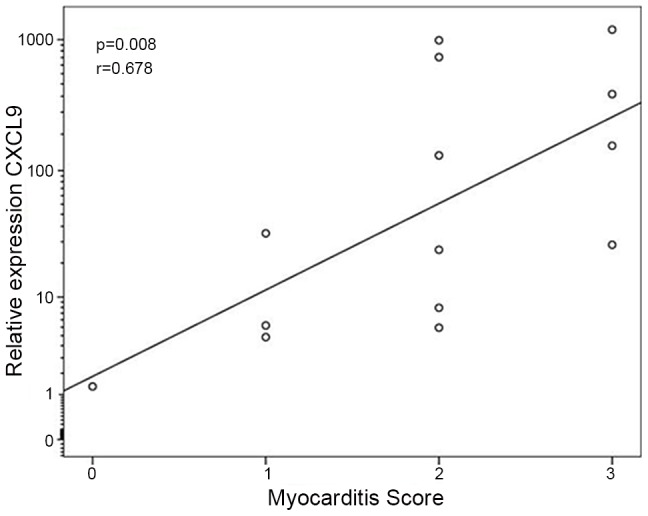
Correlation between histopathologically assessed intensity of myocarditis and mRNA expression of CXCL9 in CCC myocardium. Two-tailed p value obtained with Spearman's nonparametric correlation. n = 14.

We also analyzed whether chemokine expression was associated with histopathological parameters in CCC heart tissue. We found a positive correlation between mRNA expression of CXCL9 and the histopathologically assessed intensity of myocarditis (Figure 3). CXCL9 also showed a positive correlation with chemokine receptors CXCR3, CCR4 and CCR5, as well as chemokines CCL5, CXCL10 and CCL17. In addition, positive correlations were also found between CXCR3 and CCR5; the chemokine receptors and several of their ligands; and between several CC and CXC chemokines (Figure S2).

Given the focal nature of the myocarditis and fibrosis in CCC myocardium, we assessed the gene expression profile in different sites of the left ventricular free wall of three patients. This profile was found to be essentially concordant in samples from different sites of the myocardium of the same patients (Table S3). We also found a positive correlation of global gene expression between almost all CCC samples (Figure S3), indicating that most CCC patients share a similar pattern of gene expression, with individual-specific variation in global magnitudes of expression. These global magnitudes of gene expression seem thus to be maintained in distinct regions of the myocardium (Table S3).

### Genotypes of SNPs of CCR5, CXCL9 and CXCL10 are associated with CCC severity

In order to investigate whether the observed variation in global magnitudes of gene expression was due to genetic polymorphisms, we evaluated single nucleotide polymorphisms in the most highly expressed genes in CCC heart tissue (fold expression >10 as compared to normal donor samples). We compared genotypic and allele frequencies in the CCC and ASY groups of a sample of 325 Brazilian chronic Chagas disease patients, and also stratified CCC patients according to their LVEF status, comparing the two CCC groups according to severity of ventricular dysfunction: those patients classified as severe (LVEF≤40%) or moderate (LVEF>40%) CCC. All the SNPs were in HWE in both CCC and ASY subjects.

There are no significant differences in genotype and allele frequencies of SNP *CXCL9* rs10336 between CCC and ASY (Table 2). However, the *CXCL9* rs10336 *CC* genotype was significantly less frequent among the severe CCC than in the moderate CCC group (OR, 0.47 [95% CI, 0.25–0.87] Table 2). Accordingly, the *CXCL9* rs10336 *C* allele was significantly less frequent among the severe CCC than in the moderate CCC group. Thus, both the *C* allele and the *CC* genotype are associated to a lower risk for severe CCC.

**Table 2:** Genotype and allele frequencies for the *CXCL9* rs10336 polymorphism in patients with Chagas disease.

		CCC					
	ASY	Moderate	Severe	All			
*CXCL9* (rs10336)	(n = 146)	(n = 79)	(n = 95)	(n = 174)	*x^2^*	p	OR(95%CI)
Genotype							
TT	13(9)	4(5)	10(10)	14(8)			
TC	71(49)	36(46)	55(58)	91(52)			
CC	62(42)	39(49)	30(32)	69(40)			
Genotype comparison							
TT plus TC vs. CC							
ASY vs. CCC					0.25	0.61	0.89 (0.56–1.39)
LVEF>40% vs. LVEF≤40%					5.70	0.01*	0.47(0.25–0.87)
Allele							
T	97(33)	44(28)	75(39)	119(34)			
C	195(67)	114(72)	115(61)	229(66)			
Allele comparison T vs. C							
ASY vs. CCC					0.06	0.79	0.95(0.68–1.33)
LVEF>40% vs. LVEF≤40%					5.81	0.002*	0.59(0.37–0.93)

CCC patients were further stratified by left ventricular ejection fraction values.

Data are no. (%) of patients. Moderate CCC has LVEF>40%. Severe CCC has LVEF≤40%. CI, confidence interval. OR, odds ratio.

There are no significant differences in *GG* and *CG* frequencies of SNP *CXCL10* rs3921 polymorphism in the patients with CCC when compared with ASY patients (Table 3). However, the *CXCL10* rs3921 *GG* genotype was significantly less frequent among the severe CCC than in the moderate CCC group (OR, 0.41 [95% CI, 0.22–0.79] Table 3). Accordingly, the *CXCL10* rs3921 *G* allele was significantly less frequent among the severe CCC than in the moderate CCC group. Therefore, both the *G* allele and the *GG* genotype apparently are associated to a lower risk for severe CCC. Conversely, the *CXCL10* rs3921 *C* allele was significantly more frequent between the severe CCC than in the moderate CCC group, thus the CC genotype is associated to a higher risk for severe CCC.

**Table 3:** Genotype and allele frequencies for the *CXCL10 rs3921* polymorphism in patients with Chagas disease.

		CCC					
	ASY	Moderate	Severe	All			
*CXCL10* (rs3921)	(n = 149)	(n = 77)	(n = 93)	(n = 170)	*x^2^*	p	OR(95% CI)
Genotype							
CC	15(10)	4(5)	14(15)	18(11)			
CG	75(50)	35(45)	52(56)	87(51)			
GG	59(40)	38(50)	27(29)	65(38)			
Genotype comparison							
CC plus CG vs. GG							
ASY vs. CCC					0.06	0.80	0.94 (0.60–1.48)
LVEF>40% vs. LVEF≤40%					7.36	0.006*	0.41(0.22–0.79)
GG plus CG vs. CC							
ASY vs. CCC					0.02	0.87	1.05 (0.51–2.18)
LVEF>40% vs. LVEF≤40%					#	0.04*	3.23(1.01–10.28)
Allele							
C	105(35)	43(28)	80(43)	123(36)			
G	193(65)	111(72)	106(57)	217(64)			
Allele comparison C vs. G							
ASY vs. CCC					0.06	0.80	0.95(0.69–1.32)
LVEF>40% vs. LVEF≤40%					8.30	0.003*	0.51(0.32–0.81)

CCC patients were further stratified by left ventricular ejection fraction values.

Data are no. (%) of patients. Moderate CCC has LVEF>40%. Severe CCC has LVEF≤40%. CI, confidence interval. OR, odds ratio.

The genotype and allele frequencies of the *CCR5* rs1799988 *CT* polymorphism are given in Table 4. The genotype and allele distribution of this polymorphism in the CCC patients was not significantly different from that found among ASY patients. The *CCR5* rs1799988 *CC* genotype was significantly more frequent among the severe CCC than in the moderate CCC group (OR, 2.31 [95% CI, 1.14–4.67] Table 4). Accordingly, the *CCR5* rs1799988 *C* allele was significantly more frequent among the severe CCC than in the moderate CCC group. Results indicate that both the *C* allele and the *CC* genotype of *CCR5* rs1799988 are associated to a higher risk for severe CCC.

**Table 4:** Genotype and allele frequencies for the *CCR5* rs1799988 polymorphism in patients with Chagas disease.

		CCC					
	ASY	Moderate	Severe	All			
*CCR5* (rs1799988)	(n = 150)	(n = 78)	(n = 93)	(n = 171)	*x^2^*	p	OR(95%CI)
Genotype							
CC	45(30)	15(19)	33(35)	48(28)			
CT	63(42)	43(55)	46(49)	89(52)			
TT	42(28)	20(26)	14(15)	34(20)			
Genotype comparison							
CC plus CT vs. TT							
ASY vs. CCC					2.91	0.08	0.63(0.38–1.07)
LVEF>40% vs. LVEF≤40%					2.98	0.08	0.51(0.23–1.10)
TT plus CT vs. CC							
ASY vs. CCC					0.14	0.70	0.91 (0.56–1.47)
LVEF>40% vs. LVEF≤40%					5.55	0.01*	2.31(1.14–4.67)
Allele							
C	153(51)	73(47)	112(60)	185(54)			
T	147(49)	83(53)	74(40)	157(46)			
Allele comparison C vs. T							
ASY vs. CCC					0.61	0.61	0.88(0.64–1.20)
LVEF>40% vs. LVEF≤40%					6.15	0.01*	0.58(0.37–0.89)

CCC patients were further stratified by left ventricular ejection fraction values.

Data are no. (%) of patients. Moderate CCC has LVEF>40%. Severe CCC has LVEF≤40%. CI, confidence interval. OR, odds ratio.

No differences in genotypic and allelic frequencies were found in the studied SNPs in *CCL4*, *CCL5*, *CXCR3*, *CCL17* and *CCL19* between CCC and ASY patients (Tables S4, S5, S6, S7 and S8).

### Protective CXCL9 and CXCL10 polymorphisms are associated with lower myocardial chemokine expression and myocarditis

**Figure 4:**
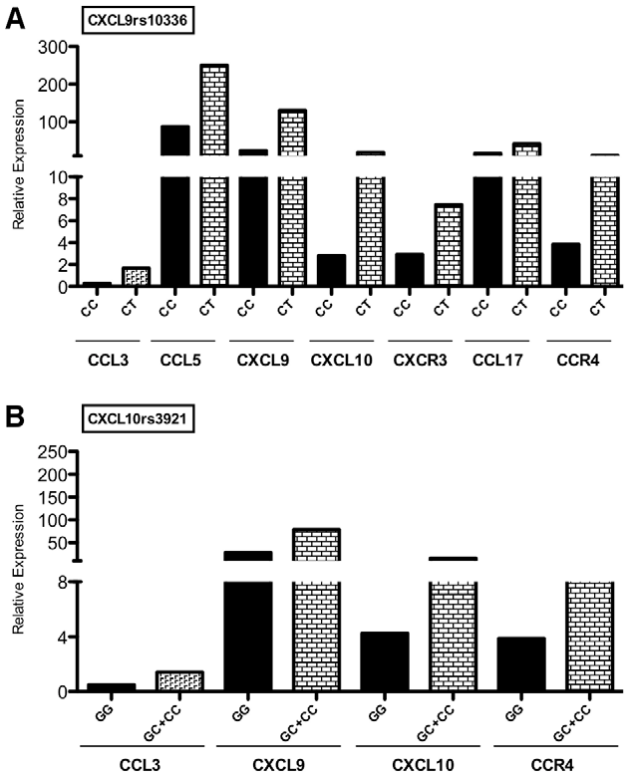
Chemokine and chemokine receptor expression in myocardium from patients bearing the *CXCL9* and *CXCL10* SNPs. Expression of chemokines and chemokine receptors in myocardium samples from patients bearing the *CXCL9* rs10336 SNP (A). Expression of chemokines and chemokine receptors in myocardium samples from patients bearing *CXCL10* rs3921 SNP (B). Real-time qPCR analysis of mRNA levels expression in myocardium samples from patients bearing the *CXCL9* rs10336 *CC* (n = 5) or *CT* (n = 9) genotypes (A) and *CXCL10* rs3921 *GG* (n = 5) or *CC* or *CG* (n = 9). The bar stands for the median.

We next analyzed whether *CXCL9* and *CXCL10* polymorphisms associated to decreased or increased risk of CCC development conveyed different levels of chemokine/chemokine receptor expression in CCC heart samples. The study could not be done for *CCR5* because there was only one *TT* patient. We found that myocardial expression of CXCL9 mRNA among samples from patients bearing the protective SNP *CXCL9* rs10336 genotype *CC* (n = 5) was 5-fold lower than that of patients bearing the *TT* or *CT* genotypes. We also found that CCL3, CCL5, CXCL10, CXCR3, CCL17 and CCR4 were 2–6-fold less expressed in SNP *CXCL9* rs10336 genotype *CC* as compared with samples from patients bearing the *TT* or *CT* genotypes (Figure 4A).

Likewise, we found that myocardial CXCL10 expression among samples from patients bearing the protective SNP *CXCL10* rs3921 genotype *GG* (n = 4) was 3-fold less intense than that of patients bearing the *CC* or *CG* genotypes. We also found that CCL3, CXCL9 and CCR4 were 2–3 fold less expressed in samples from patients carrying SNP *CXCL10* rs3921 genotype *GG* as compared with those from patients bearing the *CC* or *CG* (Figure 4B).

## Discussion

Although the pathogenic role of myocarditis in CCC is well established, the factors that lead to the maintenance of the myocardial infiltrate have remained unclear. In this paper, we report that mononuclear cells expressing CD3, CD4, CD8, CCR5, CXCR3, CCR4, CXCL9 and CCL5 are found in CCC myocardial tissue. We also found increased mRNA expression of CXCL9, CCL5, the above mentioned chemokine receptors and several of their other chemokine ligands. CXCL9 expression correlated with the intensity of the myocardial infiltrate, as well as with the mRNA expression of CXCR3, CCR5, CCR4, CCR7 and several of their ligands. The closely linked *CXCL9* rs10336 CC and *CXCL10* rs3921 *GG* genotypes were associated with protection from severe CCC, while *CCR5* rs1799988 *CC* is associated with increased risk for development of severe CCC. Myocardium samples carrying the protective *CXCL9* rs10336 *CC* genotype expressed significantly less mRNA encoding CXCL9 itself, along with CCL3, CCL5, CXCL10, CXCR3, CCL17 and CCR4, than samples carrying other *CXCL9* rs10336 genotypes. Similarly, samples carrying the protective *CXCL10* rs3921 *GG* genotype expressed less mRNA encoding CCL3, CXCL9, CXCL10 and CCR4 than samples carrying other *CXCL10* rs3921 genotypes. These results suggest that CXCL9 (and possibly CXCL10) may play an important role modulating the CXCR3/CCR5 chemokine axis and the intensity of mononuclear cell migration to CCC myocardium.

The identification of CCR5^+^ and CXCR3^+^ mononuclear cells, corroborated by the increased mRNA expression of the same chemokine receptors (Figure 1 and 2) in CCC myocardium, is in line with the prominent finding of mononuclear cells secreting IFN-γ and TNF-α in CCC heart tissue [13], [14], [38], and together are consistent with the influx of Th1 cells expressing these receptors. CCR5 is known to be expressed by macrophages, T and B cells, while CXCR3 is expressed by T and B cells; such cells may thus have been attracted by the respective chemokine ligands [39]. Indeed, CCC patients display increased numbers of CD4^+^ and CD8^+^ T cells coexpressing CXCR3/CCR5 and IFN-γ/TNF-α in peripheral blood when compared to *T. cruzi*-seropositive ASY/IND individuals [26]. In addition, CXCR3 expression was also observed in stromal cells from CCC myocardium (Figure 1B), in line with reports of CXCR3 expression in non-lymphoid cells [40], which may have contributed to the increased CXCR3 mRNA expression in CCC heart tissue. The high expression of CCR4 and CCL17 (Figure 2) is consistent with the presence of Th2 T cells [41]. However, it is unlikely that such cells are functional Th2 T cells, since preliminary observations indicate that IL-4, IL-5, and IL-13 are not expressed in CCC heart tissue (unpublished results). It is possible that other cell types, such as endothelial cells or regulatory T cells, may be responsible for CCR4 and CCL17 expression in CCC myocardium [42], [43]. The finding that CCR7 and its ligands CCL19 and CCL21 were upregulated in CCC myocardium is consistent with a role in the recruitment of CCR7^+^ cells from the periphery (Figure 2B). CCR7 can be expressed by naïve or central memory T cells, along with endothelial cells, fibroblasts and B cells [44]. Endothelial expression of CCR7 ligands is a known chemoattractant for CCR7^+^ central memory and even naïve T cells for migration into inflamed tissue [44]. CCL21, whose mRNA expression is upregulated in myocardium samples of both CCC and NIC patients, is also a chemoattractant for CCR7^+^ fibrocytes [45], inducing expression of type I collagen in vitro [46] and thus promotes fibrosis. CCL21 expression may thus be one of the stimuli driving myocardial fibrosis and pathological remodeling both in CCC and NIC.

The identification of CCL5^+^ and CXCL9^+^ mononuclear cells (Figure 1B), as well as the mRNA expression of these and other CCR5 and CXCR3-binding chemokines (Figure 2B) is in line with the observed accumulation of CCR5^+^ and CXCR3^+^ mononuclear cells in CCC myocardium. CXCR3 and CCR5-binding chemokines have been postulated to play a role in recruiting polarized Th1 T cells to sites of chronic inflammation [41], and most of them are also IFN-γ-inducible chemokines [47]. Interestingly, discrete staining for CCL5 was also observed in structures consistent with microvascular endothelium (Figure 1B), suggesting a role for this chemokine, and possibly other CCR5 ligands, in the migration and accumulation of Th1 cells in CCC heart tissue. CCL3, CCL4, CCL5, CXCL9 and CXCL10 are produced by stimulated T lymphocytes, monocytes/macrophages, as well as fibroblasts, endothelial cells and cardiomyocytes [39], [48], [49], [50] and the myocardial tissue of *T. cruzi*-infected mice [51], [52]. Indeed, treatment of *T. cruzi*-infected mice with Met-Rantes, a CCL5-based CCR5 antagonist, reduced the intensity of the inflammatory heart infiltrate, with little effect on parasitism [52], suggesting that CCL5-induced migration of CCR5 inflammatory cells may play a direct role in the genesis of acute *T. cruzi*-induced myocarditis. A recent study has shown that Beagle dogs presenting the cardiac form of *T. cruzi* infection presented higher myocardial mRNA expression of CXCL9 and CCL5 than uninfected animals, during the acute and chronic phases, respectively [53]. Moreover, microarray analysis have identified genes related to inflammation, such as chemokines, to be upregulated in myocardium of mice chronically infected with the Colombian strain of *T. cruzi* and in primary murine cardiomyocytes infected with *T. cruzi* [54], [55],

The fact that *CXCL9* mRNA expression was the only chemokine found to display significant correlation with the intensity of the myocardial infiltrate suggests CXCL9 plays a role in mononuclear cell migration to CCC myocardium. The correlation between CXCL9 expression and that of other chemokines, as well as their CXCR3/CCR5/CCR4/CCR7 receptors (Figure 3 and Supplemental data) may be partially related to the finding that CXCL9 has immunomodulatory properties, being able to directly increase expression of CCL2, CCL3 and CCL4 among other inflammatory genes such as TNF-α [56]. Expression of all three chemokines has been shown to be upregulated in CCC myocardial tissue [27], suggesting that CXCL9 may multiply its T-cell chemoattractant properties with the aid of CCR5 ligands in CCC heart tissue. It can thus be hypothesized that IFN-γ secreting type 1 T cells in CCC myocardium induce local production of CXCL9 (and perhaps CXCL10), which in turn further recruits IFN-γ-secreting CXCR3^+^/CCR5^+^ type 1 T cells and other inflammatory cells directly and indirectly, perpetuating the influx of pathogenic Th1-type T cells and inflammation.

The polymorphisms *CXCL9* rs10336 *CC* and *CXCL10* rs3921 *GG*, associated with protection from progression to severe CCC, are located in the 3′UTR region, and the polymorphism *CCR5* rs1799988 *CC*, associated with increased risk for development of severe CCC, is located at the 5′UTR region, where they may influence binding of gene expression control regions to regulatory elements. The findings are in line with previous associations of chemokine or chemokine receptor polymorphisms in CCC (CCL2 [57], CCL5 [58], CCR5 [29], [58]. However, to our knowledge, is the first study to find gene polymorphisms associated to the transition to CCC with ventricular dysfunction, which is the most clinically relevant presentation of disease, the one associated with significant morbidity and mortality. Previous gene polymorphism association studies performed in Chagas disease only disclosed polymorphisms relevant for the transition between the ASY and the CCC forms, irrespective of clinical severity (reviewed in [10], [59], [60], [61]. Associations among polymorphic variants in the *CCR5*, *CXCL9* and *CXCL10* genes and disease phenotypes have previously been reported. A sequence variant in the promoter region of the *CCR5* gene, which is associated to reduced expression of *CCR5*, is associated with protection to AIDS progression [62] and is also present at a higher frequency in ASY compared with CCC patients [29]. Although Bruck et al [35] failed to observe any association between the *CXCL9* rs10336 *CT* polymorphism and type 1 diabetes, the *CXCL9* rs2276886 *GA* polymorphic variant was associated with a reduced risk for pediatric Crohn's disease; significantly, *CXCL9* was found to be overexpressed in Crohn's disease gut tissue [63]. The *CXCL10 -201 G/A* polymorphism is associated with increased susceptibility to chronic hepatitis B disease progression in males. The *CXCL10 -201 G/*A is a functional polymorphism, where allelic variants modulate *CXCL10* expression by differential interaction with CXCL10 transcription factors [64]. Still another *CXCL10* polymorphic variant, *CXCL10* rs8878 *TT*, was found to reduce the risk of type 1 diabetes [65].

Since all our CCC myocardium samples came from clinically similar end-stage patients submitted to transplantation, it could be argued that possessing *CXCL9* and/or *CXCL10* genotypes associated to reduced myocardial chemokine expression, or even displaying a less significant inflammatory infiltrate by itself – may not be relevant for the progression of CCC. However, CCC is not a monogenic disease, and it is likely that the progression to overt inflammatory dilated cardiomyopathy may result from the combined effect and inadequate counterregulation of relevant genes. Polymorphisms in multiple innate immunity/inflammatory genes, like *IL1β*, *TNF-α*, *IL10*, *IL12*, *lymphotoxin-α*, *BAT1*, *NFKBIL1*, *MAL/TIRAP*, *MIF* have been found to associate with risk for developing CCC (reviewed in [10], [59]). In addition to interference by other genes, differential myocardial resilience, including responses to hypertrophic/fibrogenic factors occurring in CCC heart tissue (*IL1β*, *TNF-α*, *IFN-γ*, *IL18*, *CCL21*), could explain why these patients carrying the *CXCL9/CXCL10* protective genotypes progressed to end-stage cardiomyopathy. In the Syrian hamster model of chronic Chagas disease cardiomyopathy, although the intensity of chronic inflammation correlated with ventricular dilation, intensity of myocarditis was similar in hamsters dying from chronic *T. cruzi*-induced dilated cardiomyopathy and survivors euthanized 11 months post-infection [10], suggesting the existence of additional factors related to death from CCC.

The finding that the protective *CXCL9* rs10336 *CC* and *CXCL10* rs3921 *GG* genotypes were associated with lower myocardial expression of the respective chemokines indicate either that the polymorphisms are functional themselves or closely linked to functional polymorphisms. These polymorphisms are strongly linked in the caucasian and negroid populations in the HapMap and test populations (data not shown). Indeed, the *CXCL9* and *CXCL10* genes are contained in a CXC chemokine “mini-cluster’ at chromosome position 4q21.2 [61], [66] and positioned less than 20 Kb apart. The proximity and linkage of the loci renders it difficult to ascertain which of the two genes is actually responsible for the immunological effect. In fact, we found over 60 additional SNPs tightly linked to *CXCL9* rs10336 and *CXCL10* rs3921 in the reference HapMap/CEPH CEU (Northern European Caucasian) and YRI (Yoruba, African) populations in the vicinity of the CXCL9-CXCL10-CXCL11 chemokine minicluster at Chromosome 4. Some of them are at the 3′ UTR or 5′UTR regions of either CXCL9 or CXCL10, with possible transcriptional regulatory activity, and could, in theory, be responsible for the observed functional association (Table S9). The fact that samples carrying genotypes associated to lower risk also displayed reduced expression of several other chemokines and receptors, as well as with reduced intensity of the myocardial infiltrate, may suggest that the *CXCL9* and *CXCL10* genotypes control the intensity of myocarditis through modulation of CXCL9 and/or CXCL10 production and its ensuing effects on chemokine ligands of the CXCR3/CCR5/CCR4 receptors. Together with the finding of a correlation between expression of *CXCL9* and intensity of myocarditis, these results suggest that CXCL9 may be a master regulator of chemokine-driven inflammatory cell migration to CCC heart tissue. Since the intensity of the infiltrate seems to be a major pathogenic factor, inflammatory cell recruitment into the myocardium driven by CXCL9 and other CC and CXC chemokines may play an important role in the clinical progression to severe, life-threatening CCC. Several chemokine receptor antagonists are either on late clinical trials or have been licensed [67], [68]. CCR5 and CXCR3 blockers have been shown to suppress migration of inflammatory cells in acute allograft rejection [69]. Treatment with Met-Rantes ameliorated acute Chagas disease myocarditis in murine models [52]. Together with these reports, our results suggest that the CCR5/CXCR3 receptors may be therapeutic targets in CCC, where receptor antagonists could dampen inflammatory T cell migration to myocardium, thus controlling CCC myocarditis and, possibly, ventricular dysfunction and death.

## Supporting Information

Figure S1 **Presence of CD3^+^, CD4^+^, CD8^+^, CCR5^+^, CXCR3^+^, CCR4^+^, CCL5^+^ and CXCL9^+^ cells in heart tissue.** Sections were stained with primary antibodies against CD3, CD4, CD8, CCR5, CXCR3, CCR4, CCL5 and CXCL9 stained with AF633-labeled (red) and counterstained with DAPI (blue) as described in Methods.
(TIF)

Figure S2 **Correlations between the levels of expression of different genes in CCC samples.** Two-tailed nonparametric correlation of Spearman. ** p<0.01 and * p<0.05.
(TIF)

Figure S3 **Correlation between expression profiles of the 32 studied genes among different CCC myocardial samples.** Two-tailed nonparametric correlation of Spearman. ** p<0.01 and * p<0.05.
(TIF)

Table S1 **Characteristics of the primers used in gene expression analysis.**
(DOC)

Table S2 **Characteristics and Minimal Allele Frequency of tested SNPs.**
(DOC)

Table S3 **Relative expression values of 14 genes analyzed in different sites of left ventricular free wall samples from three CCC patients, CCC-7, CCC-9 and CCC-10.**
(DOC)

Table S4 **Genotype and allele frequencies for the** ***CCL4 rs1719153*** **polymorphism in patients with Chagas disease.** CCC patients were further stratified by left ventricular ejection fraction values.
(DOC)

Table S5 **Genotype and allele frequencies for the** ***CCL5 rs2107538*** **polymorphism in patients with Chagas disease.** CCC patients were further stratified by left ventricular ejection fraction values.
(DOC)

Table S6 **Genotype and allele frequencies for the** ***CXCR3 rs2280964*** **polymorphism in patients with Chagas disease.** CCC patients were further stratified by left ventricular ejection fraction values.
(DOC)

Table S7 **Genotype and allele frequencies for the** ***CCL19 rs3136658*** **polymorphism in patients with Chagas disease.** CCC patients were further stratified by left ventricular ejection fraction values.
(DOC)

Table S8 **Genotype and allele frequencies for the** ***CCL17 rs223827*** **polymorphism in patients with Chagas disease.** CCC patients were further stratified by left ventricular ejection fraction values.
(DOC)

Table S9 **SNPs in tight linkage disequilibrium with** ***CXCL9*** **rs10336 and** ***CXCL10*** **rs3921.**
(DOC)
