# Supplementary figures and images for: Myocardial Chemokine Expression and Intensity of Myocarditis in Chagas Cardiomyopathy Are Controlled by Polymorphisms in CXCL9 and CXCL10
Source: PLoS Negl Trop Dis. 2012 Oct 25;6(10):e1867. doi: 10.1371/journal.pntd.0001867 (PMC3493616; doi:10.1371/journal.pntd.0001867)

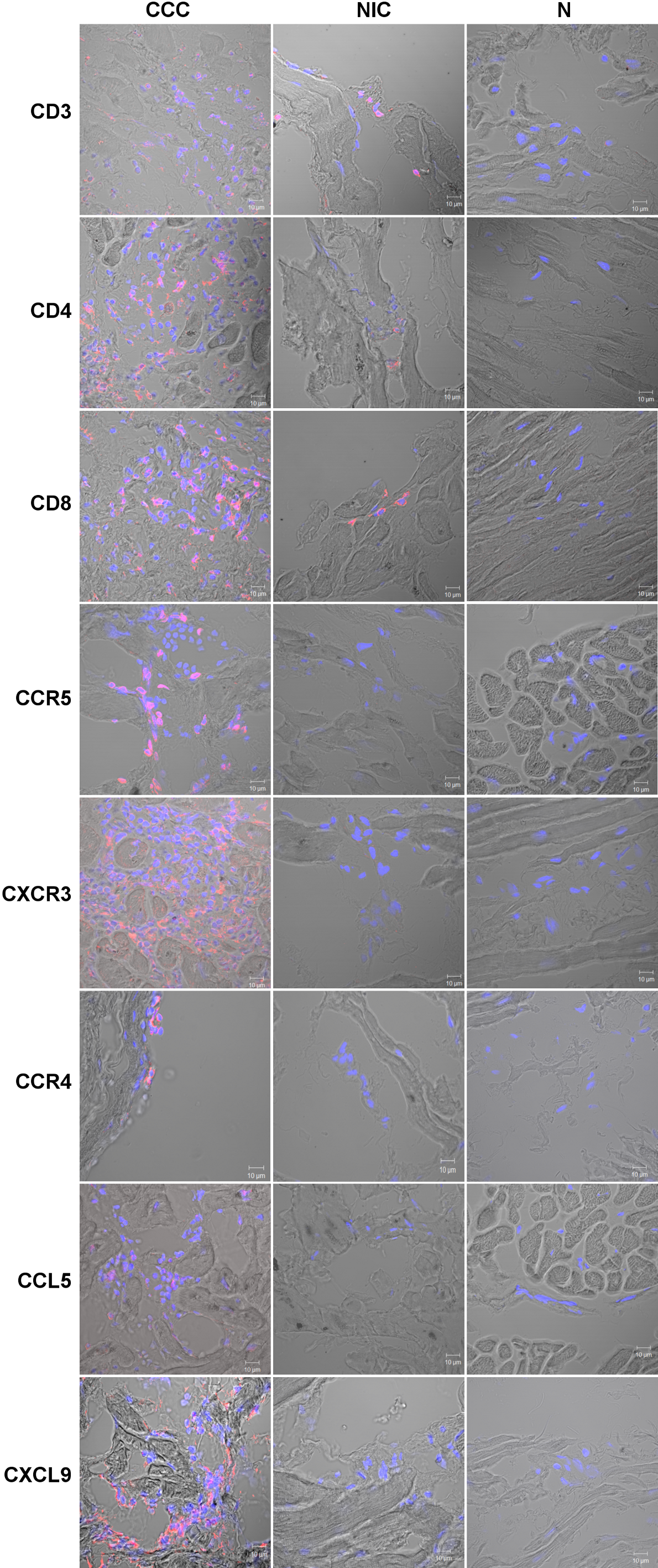

Supplement: Figure S1 — Presence of CD3+, CD4+, CD8+, CCR5+, CXCR3+, CCR4+, CCL5+ and CXCL9+ cells in heart tissue. Sections were stained with primary antibodies against CD3, CD4, CD8, CCR5, CXCR3, CCR4, CCL5 and CXCL9 stained with AF633-labeled (red) and counterstained with DAPI (blue) as described in Methods. (TIF) [file pntd.0001867.s001.tif]

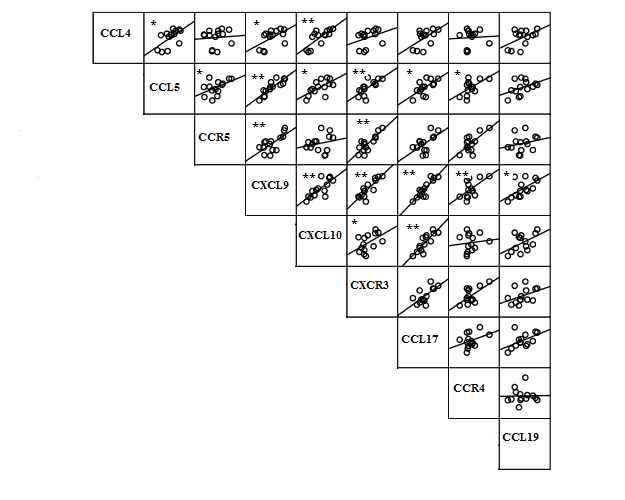

Supplement: Figure S2 — Correlations between the levels of expression of different genes in CCC samples. Two-tailed nonparametric correlation of Spearman. ** p<0.01 and * p<0.05. (TIF) [file pntd.0001867.s002.tif]

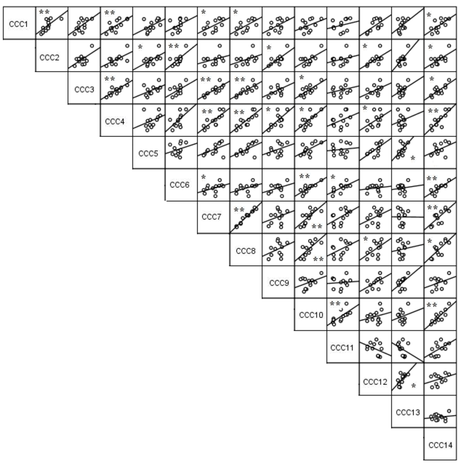

Supplement: Figure S3 — Correlation between expression profiles of the 32 studied genes among different CCC myocardial samples. Two-tailed nonparametric correlation of Spearman. ** p<0.01 and * p<0.05. (TIF) [file pntd.0001867.s003.tif]
