# Supplementary material for: Myocardial Chemokine Expression and Intensity of Myocarditis in Chagas Cardiomyopathy Are Controlled by Polymorphisms in CXCL9 and CXCL10
Source: PLoS Negl Trop Dis. 2012 Oct 25;6(10):e1867. doi: 10.1371/journal.pntd.0001867 (PMC3493616; doi:10.1371/journal.pntd.0001867)
Supplement: Table S1 — Characteristics of the primers used in gene expression analysis. (DOC) [file pntd.0001867.s004.doc]

**Table S1**. Characteristics of the primers used in gene expression analysis.

| Gene Name | Gene bank accession number | Sequence 5’→ 3’ | Amplicon (pb) | Tm Amplicon (ºC) |
| --- | --- | --- | --- | --- |
| *CCL1* | M57502 | GCTCCAATGAGGGCTTAATATTCA | 91 | 79 |
|  |  | ATTTTTCTGTGCCTCTGAACCCAT |  |  |
| *CCL3* | AF043339 | ACCAGTTCTCTGCATCACTTGCT  GCTGCTCGTCTCAAAGTAGTCAGC | 110 | 82 |
| *CCL4* | J04130 | GCTTCCTCGCAACTTTGTGGT  CACTGGGATCAGCACAGACTTG | 110 | 80 |
| *CCL5* | M21121 | CGTGCCCACATCAAGGAGTATT  CACACACTTGGCGGTTCTTTC | 91 | 80 |
| *CCL17* | D43767 | CACATCCACGCAGCTCGA  TGGTACCACGTCTTCAGCTTTCTA | 98 | 81 |
| *CCL19* | NM_006274 | GCCTGCTGGTTCTCTGGACTT  TTTCTGGGTCACAGACAGGCA | 86 | 81 |
| *CCL21* | NM_002989 | TCCATCCCAGCTATCCTGTTCT  TGTCTTGTCCATGCTGCATCA | 108 | 83 |
| *CCL22* | U83171 | CTGCGCGTGGTGAAACACTT  CACAGATCTCCTTATCCCTGAAGGT | 91 | 81 |
| *CXCL9* | X72755 | TCTGATTGGAGTGCAAGGAACC  GGTCTTTCAAGGATTGTAGGTGGA | 98 | 79 |
| *CXCL10* | X02530 | TCCACGTGTTGAGATCATTGCTA  GCTTTCAGTAAATTCTTGATGGCC | 93 | 74 |
| *CCR4* | X85740 | CCCTTCCTGGCTTTCTGTTCA  TTCCACGTCGTGGAGTTGAGA | 91 | 78 |
| *CCR5* | U57840 | TCCGCTCTACTCACTGGTGTTCA  CATGCTCTTCAGCCTTTTGCAG | 91 | 78 |
| *CCR7* | L31581 | CTCTCCTTGTCATTTTCCAGGTATG  TTGGAGCACAAAGACTCGAACA | 109 | 79 |
| *CCR8* | NM_005201 | ATGCCCTAAAGGTGAGGACGAT  ACTAGCAATGGGATGGTAGCCA | 91 | 80 |
| *CXCR3* | NM_001504 | GTCCTTGAGGTGAGTGACCACC  ACGAGTCACTCTCGTTTTCTCCA | 106 | 80 |
| *ANP* | NM_006172 | GGTCAGACCAGAGCTAATCCCA  TCTTTTCTTCCAAATGGTCCAGC | 91 | 78 |
| *BNP* | NM_002521 | TGCTCTTCTTGCATCTGGCTTT  GGTTGCGCTGCTCCTGTAAC | 105 | 85 |
| *GAPDH* | NM_002046 | TGGTCTCCTCTGACTTCA  AGCCAAATTCGTTGTCAT | 117 | 82 |
