# Supplementary material for: Myocardial Chemokine Expression and Intensity of Myocarditis in Chagas Cardiomyopathy Are Controlled by Polymorphisms in CXCL9 and CXCL10
Source: PLoS Negl Trop Dis. 2012 Oct 25;6(10):e1867. doi: 10.1371/journal.pntd.0001867 (PMC3493616; doi:10.1371/journal.pntd.0001867)
Supplement: Table S2 — Characteristics and Minimal Allele Frequency of tested SNPs. (DOC) [file pntd.0001867.s005.doc]

**Table S2.** Characteristics and Minimal Allele Frequency of tested SNPs.

| Gene | SNP | Region | Chromossome | Gene location | HapMap | | Reference |
| --- | --- | --- | --- | --- | --- | --- | --- |
|  |  |  |  |  | Population | Genotype (MAF Frequency) |  |
| CCL4 | rs1719153 | 3’UTR | Chr.17 | 34433229 | CEU  CHB  JPT  YRI | AA(0.48) AT(0.46) TT(0.05)  AA(0.54) AT(0.38) TT(0.06)  AA(0.50) AT(0.43) TT(0.06)  AA(0.98) AT(0.01) TT(0) | [70] |
| CCL5 | rs2107538 | 5’UTR | Chr. 17 | 34207780 | CEU  CHB  JPT  YRI | CC(0.81) CT(0.19) TT(0)  CC(0.44) CT(0.42) TT(0.13)  CC(0.36) CT(0.47) TT(0.15)  CC(0.33) CT(0.50) TT(0.15) | [70] |
| CCL17 | rs223827 | Intron | Chr. 16 | 57446007 | CEU  CHB  JPT  YRI | CC(0.13) CT(0.59) TT(0.27)  CC(0.28) CT(0.41) TT(0.30)  CC(0.35) CT(0.52) TT(0.11)  CC(0.49) CT(0.38) TT(0.12) | unpublished |
| CCL19 | rs3136658 | Intron | Chr. 9 | 34690400 | CEU  CHB  JPT  YRI | GG(0.68) AG(0.30) AA(0.17)  GG(0.84) AG(0.15) AA(0)  GG(0.90) AG(0.09) AA(0)  GG(1.00) AG(n/a) AA(0) | unpublished |
| CXCL9 | rs10336 | 3’UTR | Chr. 4 | 76922988 | CEU  CHB  JPT  YRI | TT(0.25) CT(0.48) CC(0.25)  TT(0) CT(0.08) CC(0.91)  TT(0) CT(0.09) CC(0.90)  TT(0.12) CT(0.32) CC(0.55) | [35] |
| CXCL10 | rs3921 | 3’UTR | Chr. 4 | 76942943 | CEU  CHB  JPT  YRI | GG(0.28) CG(0.47) CC(0.24)  GG(0) CG(0.08) CC(0.91)  GG(0) CG(0.09) CC(0.90)  GG(0.12) CG(0.37) CC(0.50) | [35] |
| CCR5 | rs1799988 | 5’UTR | Chr. 3 | 46412259 | CEU  CHB  JPT  YRI | CC(-) CT(-) TT(-)  CC(-) CT(-) TT(-)  CC(-) CT(-) TT(-)  CC(-) CT(-) TT(-) | [36] |
| CXCR3 | rs2280964 | Intron | Chr. X | 70838054 | CEU  CHB  JPT  YRI | CC(0.58) CT(0.17) TT(0.24)  CC(0.58) CT(0.12) TT(0.29)  CC(0.63) CT(0.73) TT(0.29)  CC(0.76) CT(0.07) TT(0.16) | unpublished |

MAF: Minimal Allele Frequency. CEU: CEPH (Utah residents with ancestry from northern and western Europe). CHB: Han Chinese in Beijing, China. JPT: Japanese in Tokyo, Japan. YRI: Yoruba in Ibadan, Nigeria.
