# Supplementary material for: Myocardial Chemokine Expression and Intensity of Myocarditis in Chagas Cardiomyopathy Are Controlled by Polymorphisms in CXCL9 and CXCL10
Source: PLoS Negl Trop Dis. 2012 Oct 25;6(10):e1867. doi: 10.1371/journal.pntd.0001867 (PMC3493616; doi:10.1371/journal.pntd.0001867)
Supplement: Table S3 — Relative expression values of 14 genes analyzed in different sites of left ventricular free wall samples from three CCC patients, CCC-7, CCC-9 and CCC-10. (DOC) [file pntd.0001867.s006.doc]

**Table S3**. Relative expression values of 14 genes analyzed in different sites of left ventricular free wall samples from three CCC patients, CCC-7, CCC-9 and CCC-10.

| Gene | Samples | | | | | | |
| --- | --- | --- | --- | --- | --- | --- | --- |
|  | CCC-7 | | | CCC-9 | | CCC-10 | |
|  | Site 1 | Site 2 | Site 3 | Site 1 | Site 2 | Site 1 | Site 2 |
| CCL3 | 1.0 | 0.6 | 0.3 | 0.3 | 0.2 | 2.9 | 3.1 |
| CCL4 | 22.6 | 16.6 | 16.3 | 11.5 | 7.6 | 17.0 | 22.5 |
| CCL5 | 199.8 | 237.6 | 136.7 | 60.2 | 0.2 | 808.3 | 1405.2 |
| CCR5 | 52.2 | 6.5 | 17.5 | 19.6 | 4.1 | 1.7 | 10.8 |
| CXCL9 | 384.2 | 236.3 | 249.7 | 8.1 | 4.4 | 130.6 | 203.8 |
| CXCL10 | 60.9 | 35.6 | 23.5 | 4.3 | 1.6 | 27.6 | 24.3 |
| CXCR3 | 57.9 | 33.5 | 31.5 | 1.2 | 3.2 | 7.4 | 7.5 |
| CCL17 | 171.6 | 149.0 | 134.0 | 12.6 | 6.3 | 47.4 | 36.8 |
| CCR4 | 3.3 | 4.0 | 4.1 | 17.0 | 2.1 | 4.5 | 10.8 |
| CCR8 | 0.5 | 0.3 | 0.2 | 16.3 | 0.4 | 0.43 | 0.2 |
| CCL19 | 598.4 | 118.9 | 140.0 | 7.6 | 4.1 | 25.1 | 60.0 |
| CCL21 | 4.3 | 4.4 | 5.6 | 1.3 | 3.4 | 5.2 | 12.3 |
| ANP | 15.1 | 43.9 | 27.3 | 155.2 | 8.5 | 88.1 | 153.4 |
| BNP | 90.8 | 235.8 | 121.0 | 828.2 | 78.6 | 832.6 | 756.8 |

“-“ undetectable gene expression
