# Supplementary material for: Myocardial Chemokine Expression and Intensity of Myocarditis in Chagas Cardiomyopathy Are Controlled by Polymorphisms in CXCL9 and CXCL10
Source: PLoS Negl Trop Dis. 2012 Oct 25;6(10):e1867. doi: 10.1371/journal.pntd.0001867 (PMC3493616; doi:10.1371/journal.pntd.0001867)
Supplement: Table S4 — Genotype and allele frequencies for the CCL4 rs1719153 polymorphism in patients with Chagas disease. CCC patients were further stratified by left ventricular ejection fraction values. (DOC) [file pntd.0001867.s007.doc]

**Table S4.** Genotype and allele frequencies for the *CCL4 rs1719153* polymorphism in patients with Chagas disease. CCC patients were further stratified by left ventricular ejection fraction values.

|  |  |  | CCC |  |  |  |  |
| --- | --- | --- | --- | --- | --- | --- | --- |
|  | ASY | All | Moderate | Severe |  |  |  |
| CCL4 (rs1719153) | (n=151) | (n=174) | (n=79) | (n=95) | *x^2^* | p | OR(95%CI) |
| Genotype |  |  |  |  |  |  |  |
| AA | 93(62) | 97(56) | 50(63) | 47(49) |  |  |  |
| AT | 49(32) | 72(41) | 25(32) | 47(49) |  |  |  |
| TT | 9(6) | 5(3) | 4(5) | 1(2) |  |  |  |
| Genotype comparison |  |  |  |  |  |  |  |
| AA plus AT vs. TT |  |  |  |  |  |  |  |
| ASY vs. CCC |  |  |  |  | # | 0.18 | 0.46(0.15-1.42) |
| LVEF>40% vs. LVEF ≤ 40% |  |  |  |  | # | 0,17 | 0,19(0,02-1,82) |
| TT plus AT vs. AA |  |  |  |  |  |  |  |
| ASY vs. CCC |  |  |  |  | 1.13 | 0.28 | 0.78 (0.50-1.22) |
| LVEF>40% vs. LVEF ≤ 40% |  |  |  |  | 3.33 | 0,06 | 0,56(0.30-1.04) |
| Allele |  |  |  |  |  |  |  |
| A | 235(78) | 266(76) | 125(79) | 141(74) |  |  |  |
| T | 67(22) | 82(24) | 33(21) | 49(26) |  |  |  |
| Allele comparison A vs. T |  |  |  |  |  |  |  |
| ASY vs. CCC |  |  |  |  | 0.17 | 0,67 | 1.08(0,74-1,56) |
| LVEF>40% vs. LVEF≤40% |  |  |  |  | 1.15 | 0,28 | 1.31(0,79-1.17) |

Data are no. (%) of patients. Moderate CCC has LVEF > 40%. Severe CCC has LVEF ≤ 40%. CI, confidence interval. OR, odds ratio.
