# Supplementary material for: Myocardial Chemokine Expression and Intensity of Myocarditis in Chagas Cardiomyopathy Are Controlled by Polymorphisms in CXCL9 and CXCL10
Source: PLoS Negl Trop Dis. 2012 Oct 25;6(10):e1867. doi: 10.1371/journal.pntd.0001867 (PMC3493616; doi:10.1371/journal.pntd.0001867)
Supplement: Table S5 — Genotype and allele frequencies for the CCL5 rs2107538 polymorphism in patients with Chagas disease. CCC patients were further stratified by left ventricular ejection fraction values. (DOC) [file pntd.0001867.s008.doc]

**Table S5.** Genotype and allele frequencies for the *CCL5 rs2107538* polymorphism in patients with Chagas disease,.CCC patients were further stratified by left ventricular ejection fraction values.

|  |  |  | CCC |  |  |  |  |
| --- | --- | --- | --- | --- | --- | --- | --- |
|  | ASY | All | Moderate | Severe |  |  |  |
| CCL5 (rs2107538) | (n=146) | (n=167) | (n=77) | (n=90) | *x^2^* | p | OR(95%CI) |
| Genotype |  |  |  |  |  |  |  |
| CC | 72(49) | 91(54) | 41(54) | 50(56) |  |  |  |
| CT | 60(41) | 64(38) | 31(40) | 33(37) |  |  |  |
| TT | 14(10) | 12(7) | 5(6) | 7(8) |  |  |  |
| Genotype comparison |  |  |  |  |  |  |  |
| CC plus CT vs. TT |  |  |  |  |  |  |  |
| ASY vs. CCC |  |  |  |  | 0.59 | 0.44 | 0.73(0.32-1.63) |
| LVEF>40% vs. LVEF ≤ 40% |  |  |  |  | # | 1.00 | 1.21(0,36-3.99) |
| TT plus CT vs. CC |  |  |  |  |  |  |  |
| ASY vs. CCC |  |  |  |  | 0.82 | 0.36 | 1.23 (0.78-1.92) |
| LVEF>40% vs. LVEF ≤ 40% |  |  |  |  | 0.08 | 0,76 | 1.09(0.59-2.02) |
| Allele |  |  |  |  |  |  |  |
| C | 204(70) | 246(74) | 113(73) | 133(74) |  |  |  |
| T | 88(30) | 88(26) | 41(27) | 47(26) |  |  |  |
| Allele comparison C vs. T |  |  |  |  |  |  |  |
| ASY vs. CCC |  |  |  |  | 1.10 | 0,29 | 0.82(0,58-1,17) |
| LVEF>40% vs. LVEF≤40% |  |  |  |  | 0.01 | 0,91 | 0.97(0,59-1.58) |

Data are no. (%) of patients. Moderate CCC has LVEF > 40%. Severe CCC has LVEF ≤ 40%. CI, confidence interval. OR, odds ratio.
