# Supplementary material for: Myocardial Chemokine Expression and Intensity of Myocarditis in Chagas Cardiomyopathy Are Controlled by Polymorphisms in CXCL9 and CXCL10
Source: PLoS Negl Trop Dis. 2012 Oct 25;6(10):e1867. doi: 10.1371/journal.pntd.0001867 (PMC3493616; doi:10.1371/journal.pntd.0001867)
Supplement: Table S6 — Genotype and allele frequencies for the CXCR3 rs2280964 polymorphism in patients with Chagas disease. CCC patients were further stratified by left ventricular ejection fraction values. (DOC) [file pntd.0001867.s009.doc]

**Table S6.** Genotype and allele frequencies for the *CXCR3 rs2280964* polymorphism in patients with Chagas disease. CCC patients were further stratified by left ventricular ejection fraction values.

|  |  |  | CCC |  |  |  |  |
| --- | --- | --- | --- | --- | --- | --- | --- |
|  | ASY | All | Moderate | Severe |  |  |  |
| CXCR3 (rs2280964) | (n=64) | (n=88) | (n=30) | (n=58) | *x^2^* | p | OR(95%CI) |
| Genotype |  |  |  |  |  |  |  |
| CC | 45(70) | 73(83) | 27(90) | 46(92) |  |  |  |
| TT | 19(30) | 15(17) | 3(10) | 12(24) |  |  |  |
| Genotype comparison |  |  |  |  |  |  |  |
| CC vs. TT |  |  |  |  |  |  |  |
| ASY vs. CCC |  |  |  |  | 3.41 | 0.06 | 0.48(0.22-1.05) |
| LVEF>40% vs. LVEF ≤ 40% |  |  |  |  | # | 0.24 | 2.34(0.60-9.07) |
| Allele |  |  |  |  |  |  |  |
| C | 90(70) | 146(83) | 54(90) | 92(79) |  |  |  |
| T | 38(30) | 30(17) | 6(10) | 24(21) |  |  |  |
| Allele comparison C vs. T |  |  |  |  |  |  |  |
| ASY vs. CCC |  |  |  |  | 6.82 | 0.009* | 0.48(0.28-0.84) |
| LVEF>40% vs. LVEF≤40% |  |  |  |  | 3.19 | 0.07 | 2.34(0.90-6.10) |

Data are no. (%) of patients. Moderate CCC has LVEF > 40%. Severe CCC has LVEF ≤ 40%. CI, confidence interval. OR, odds ratio.
