# Supplementary material for: Myocardial Chemokine Expression and Intensity of Myocarditis in Chagas Cardiomyopathy Are Controlled by Polymorphisms in CXCL9 and CXCL10
Source: PLoS Negl Trop Dis. 2012 Oct 25;6(10):e1867. doi: 10.1371/journal.pntd.0001867 (PMC3493616; doi:10.1371/journal.pntd.0001867)
Supplement: Table S7 — Genotype and allele frequencies for the CCL19 rs3136658 polymorphism in patients with Chagas disease. CCC patients were further stratified by left ventricular ejection fraction values. (DOC) [file pntd.0001867.s010.doc]

**Table S7.** Genotype and allele frequencies for the CCL19 rs3136658 polymorphism in patients with Chagas disease. CCC patients were further stratified by left ventricular ejection fraction values.

|  |  |  | CCC |  |  |  |  |
| --- | --- | --- | --- | --- | --- | --- | --- |
|  | ASY | All | Moderate | Severe |  |  |  |
| CCL19 (rs3136658) | (n=150 | (n=171) | (n=77) | (n=94) | x2 | p | OR(95%CI) |
| Genotype |  |  |  |  |  |  |  |
| GG | 110(74) | 134(81) | 61(80) | 73(82) |  |  |  |
| GA | 37(25) | 30(18) | 15(20) | 15(17) |  |  |  |
| AA | 1(1) | 1(1) | 0(0) | 1(1) |  |  |  |
| Genotype comparison |  |  |  |  |  |  |  |
| GG plus GA vs. AA |  |  |  |  |  |  |  |
| ASY vs. CCC |  |  |  |  | # | 1.00 | 0.89(0.05-14.47) |
| LVEF>40% vs. LVEF ≤ 40% |  |  |  |  | # | 1.00 | 2.59(0.10-64.64) |
| AA plus GA vs. GG |  |  |  |  |  |  |  |
| ASY vs. CCC |  |  |  |  | 2.15 | 0.14 | 1.49(0.87-2.55) |
| LVEF>40% vs. LVEF ≤ 40% |  |  |  |  | 0.08 | 0.77 | 1.12(0.51-2.45) |
| Allele |  |  |  |  |  |  |  |
| G | 257(87) | 298(90) | 137(90) | 161(90) |  |  |  |
| A | 39(13) | 32(10) | 15(10) | 17(10) |  |  |  |
| Allele comparison G vs. A |  |  |  |  |  |  |  |
| ASY vs. CCC |  |  |  |  | 1.87 | 0.17 | 0.70(0,43-1.63) |
| LVEF>40% vs. LVEF≤40% |  |  |  |  | 0.009 | 0.92 | 0.96(0.46-2.00) |

Data are no. (%) of patients. Moderate CCC has LVEF > 40%. Severe CCC has LVEF ≤ 40%. CI, confidence interval. OR, odds ratio.
