# Supplementary material for: Myocardial Chemokine Expression and Intensity of Myocarditis in Chagas Cardiomyopathy Are Controlled by Polymorphisms in CXCL9 and CXCL10
Source: PLoS Negl Trop Dis. 2012 Oct 25;6(10):e1867. doi: 10.1371/journal.pntd.0001867 (PMC3493616; doi:10.1371/journal.pntd.0001867)
Supplement: Table S8 — Genotype and allele frequencies for the CCL17 rs223827 polymorphism in patients with Chagas disease. CCC patients were further stratified by left ventricular ejection fraction values. (DOC) [file pntd.0001867.s011.doc]

**Table S8.** Genotype and allele frequencies for the *CCL17 rs223827* polymorphism in patients with Chagas disease. CCC patients were further stratified by left ventricular ejection fraction values.

|  |  |  | CCC |  |  |  |  |
| --- | --- | --- | --- | --- | --- | --- | --- |
|  | ASY | All | Moderate | Severe |  |  |  |
| CCL17 (rs223827) | (n=150 | (n=171) | (n=77) | (n=94) | *x^2^* | p | OR(95%CI) |
| Genotype |  |  |  |  |  |  |  |
| CC | 42(28) | 41(24) | 16(21) | 25(27) |  |  |  |
| CT | 69(46) | 79(46) | 36(47) | 43(46) |  |  |  |
| TT | 39(26) | 51(30) | 25(32) | 26(28) |  |  |  |
| Genotype comparison |  |  |  |  |  |  |  |
| CC plus CT vs. TT |  |  |  |  |  |  |  |
| ASY vs. CCC |  |  |  |  | 0.57 | 0.44 | 1.21(0.74-1.97) |
| LVEF>40% vs. LVEF ≤ 40% |  |  |  |  | 0.46 | 0.49 | 0.79(0.41-1.53) |
| TT plus CT vs. CC |  |  |  |  |  |  |  |
| ASY vs. CCC |  |  |  |  | 0.67 | 0.41 | 0.81 (0.49-1.33) |
| LVEF>40% vs. LVEF ≤ 40% |  |  |  |  | 0.78 | 0.37 | 1.38(0.67-2.82) |
| Allele |  |  |  |  |  |  |  |
| C | 153(51) | 161(47) | 68(44) | 93(49) |  |  |  |
| T | 147(49) | 181(53) | 86(56) | 95(51) |  |  |  |
| Allele comparison C vs. T |  |  |  |  |  |  |  |
| ASY vs. CCC |  |  |  |  | 0.98 | 0.32 | 1.17(0,85-1.59) |
| LVEF>40% vs. LVEF≤40% |  |  |  |  | 0.95 | 0.32 | 0.80(0,52-1.23) |

Data are no. (%) of patients. Moderate CCC has LVEF > 40%. Severe CCC has LVEF ≤ 40%. CI, confidence interval. OR, odds ratio.
