# Supplementary material for: Myocardial Chemokine Expression and Intensity of Myocarditis in Chagas Cardiomyopathy Are Controlled by Polymorphisms in CXCL9 and CXCL10
Source: PLoS Negl Trop Dis. 2012 Oct 25;6(10):e1867. doi: 10.1371/journal.pntd.0001867 (PMC3493616; doi:10.1371/journal.pntd.0001867)
Supplement: Table S9 — SNPs in tight linkage disequilibrium with CXCL9 rs10336 and CXCL10 rs3921. (DOC) [file pntd.0001867.s012.doc]

**Table S9.** SNPs in tight linkage disequilibrium with *CXCL9* rs10336 and *CXCL10* rs3921.

| Variation Name | Gene Name | Position on Chrom. 4 (bp) | Variant Alleles | Minor Allele | Position in transcript | Linkage YRI | Linkage CEU |
| --- | --- | --- | --- | --- | --- | --- | --- |
| rs13138223 |  | 76859615 | A/T | T | 3' |  | both |
| rs6826085 |  | 76870229 | C/T | T | 3' | both | both |
| rs2242471 |  | 76878716 | G/C | C | 3' | both |  |
| rs2242473 |  | 76878956 | G/A | A | 3' | rs10336 |  |
| rs2273 |  | 76889388 | C/T | T | 3' | rs10336 |  |
| rs4859413 |  | 76898250 | C/T | C | 3' | both | both |
| rs4859581 |  | 76906974 | T/C | T | 3' |  | both |
| rs4282209 |  | 76919536 | A/T/C/G | A | 3' | both | both |
| **rs10336** | CXCL9 | 76922988 | T/C | C | 3'UTR | rs3921 | rs3921 |
| rs10031452 | CXCL9 | 76924933 | T/C | T | Intron 3 |  | both |
| rs6532083 |  | 76929201 | A/G | A | Intergenic Region |  | both |
| rs7670789 |  | 76940381 | G/A/T/C | G | Intergenic Region | both | both |
| rs10014837 |  | 76941818 | G/A | G | Intergenic Region | both | rs3921 |
| rs8878 | CXCL10 | 76942300 | A/G | A | 3'UTR | both | both |
| **rs3921** | CXCL10 | 76942943 | C/G | C | 3'UTR | rs10336 | rs10336 |
| rs4859584 | CXCL10 | 76943137 | G/C | G | Intron 3 | rs10336 |  |
| rs4859586 | CXCL10 | 76943235 | T/C | T | Intron 3 | rs3921 | both |
| rs4859587 | CXCL10 | 76943296 | A/C | A | Intron 3 | rs3922 | both |
| rs4859588 | CXCL10 | 76943677 | G/A | G | Intron 2 | both | both |
| rs4257674 |  | 76945685 | A/G | A | Intergenic Region | both | both |
| rs4417995 |  | 76945999 | T/C | T | Intergenic Region | both | both |
| rs4386624 |  | 76946139 | G/C | G | Intergenic Region |  | both |
| rs9884374 |  | 76946718 | A/T | A | Intergenic Region | both | both |
| rs4241579 |  | 76947284 | A/G | A | Intergenic Region | both | both |
| rs4859590 |  | 76948388 | A/G | A | Intergenic Region | both | both |
| rs4859591 |  | 76948578 | G/A | G | Intergenic Region | both | both |
| rs4859414 |  | 76949997 | G/A | G | Intergenic Region | rs10336 |  |
| rs4859595 |  | 76950062 | C/T | C | Intergenic Region | both | both |
| rs6844097 |  | 76950757 | T/C | T | Intergenic Region |  | both |
| rs6814012 |  | 76950778 | C/T | C | Intergenic Region |  | both |
| rs6814817 |  | 76951170 | C/T | C | Intergenic Region | rs3921 | both |
| rs6845396 |  | 76951550 | A/C | A | Intergenic Region | rs3921 | both |
| rs6850760 |  | 76951684 | T/A | T | Intergenic Region | both | both |
| rs10021768 |  | 76951764 | T/C | T | Intergenic Region | both | both |
| rs11097212 |  | 76952879 | T/C | T | Intergenic Region |  | both |
| rs4302486 |  | 76953077 | A/G | A | Intergenic Region | rs10336 |  |
| rs4512021 |  | 76954407 | A/G | A | Intergenic Region |  | both |
| rs4619915 | CXCL11 | 76955201 | A/G | A | 3'UTR |  | both |
| rs7436646 | CXCL11 | 76955229 | T/G | T | 3'UTR | both | both |
| rs10017431 | CXCL11 | 76955406 | T/C | T | 3'UTR | both | rs3921 |
| rs6532111 | CXCL11 | 76955914 | T/C | T | 3'UTR | both | both |
| rs4859415 | CXCL11 | 76956528 | A/G | A | Intron 1 | both | both |
| rs6819597 | CXCL11 | 76957171 | C/T | C | 5'UTR |  | both |
| rs6532114 | CXCL11 | 76957974 | T/A/C/G | T | 5' | both | both |
| rs7674409 | CXCL11 | 76958334 | C/G | C | 5' | both | both |
| rs4129781 | CXCL11 | 76959388 | C/G | C | 5' | rs10336 |  |
| rs6532121 |  | 76962606 | A/T | A | 5' | rs3921 | both |
| rs7684461 |  | 76962996 | T/G | T | 5' | both | both |
| rs6816898 |  | 76969057 | C/A | C | 5' | rs3921 | both |
| rs4406046 |  | 76969955 | A/G | A | 5' | both | both |
| rs6532157 |  | 76974373 | C/T | C | 5' | both | both |
| rs13128319 |  | 76975206 | G/A | G | 5' |  | both |
| rs4422436 |  | 76977784 | T/C | T | 5' | rs3921 | both |
| rs4380549 |  | 76979082 | C/T | C | 5' |  | both |
| rs4616778 |  | 76981206 | A/G | A | 5' | both | both |
| rs4333205 |  | 76984795 | A/T | A | 5' | both | both |
| rs10017484 |  | 76984936 | C/T | C | 5' | both | both |
| rs6840980 |  | 76985331 | T/C | T | 5' | both |  |
| rs5005114 |  | 76986040 | G/A | G | 5' | both | both |
| rs4859416 |  | 76986499 | A/G | A | 5' | rs3921 | both |
| rs10856875 |  | 76987167 | T/C | T | 5' | both | both |
| rs11097223 |  | 76987496 | G/A | G | 5' | rs3921 | both |
| rs4304003 |  | 76995463 | G/A | G | 5' | both | both |

A 2MB area around the chromosome 4 chemokine minicluster encompassing CXCL9, CXCL10, CXCL11 was searched for SNPs in tight linkage disequilibrium (r^2^>0.8) with CXCL9 rs10336 and CXCL10 rs3921 (boxed and bold-faced in their respective gene locations) in the reference HapMap/CEPH CEU ( Northern European Caucasian) and YRI (Yoruba, African) populations. Gray areas, boundaries of each gene. Linkage to either of the SNPs (or both) is indicated for the CEU and YRI reference populations. Blank spaces indicate no strong linkage (r^2^<0.8) with either SNP in that population.
